# Game-Based Learning and Gamification in Laparoscopic Skills Education: Meta-Analysis and Systematic Review

**DOI:** 10.2196/81331

**Published:** 2026-10-29

**Authors:** Xiangqing Meng, Zhanshuo Wan, Wei Li, Yao Lu, Meixuan Su, Youness Zidoun, Hongliang Xian, Jing Tian, Xiaobo Xie

**Affiliations:** 1 Department of Clinical Skills Training Center, Zhujiang Hospital, Southern Medical University, 253 Gongye Avenue, Haizhu District, Guangzhou, 510280, China, 86 186 6466 3051; 2 Department of Joint and Orthopedics, Orthopedic Center, Zhujiang Hospital, Southern Medical University, Guangzhou, China; 3 Clinical Research Center, Zhujiang Hospital, Southern Medical University, Guangzhou, Guangdong, China; 4 State Key Laboratory of Multi-organ Injury Prevention and Treatment, Southern Medical University, Guangzhou, Guangdong, China; 5 Department of Gastroenterology, Nanfang Hospital of Southern Medical University, Guangzhou, Guangdong, China; 6 College of Medicine, Dubai Medical University, Dubai, United Arab Emirates; 7 Division of Orthopedics and Traumatology, Department of Orthopedics, Nanfang Hospital of Southern Medical University, Guangzhou, Guangdong, China

**Keywords:** systematic review, gamification, laparoscopy, medical education, video games

## Abstract

**Background:**

Gamification, the integration of game elements into education and game-based learning, is increasingly applied to laparoscopic skills training. However, systematic evidence on its effectiveness and practical feasibility remains limited and fragmented.

**Objective:**

This study aimed to evaluate the effectiveness and feasibility of gamified interventions and game-based learning in improving laparoscopic skills education.

**Methods:**

We searched PubMed, Embase, Cochrane Library, Web of Science, Scopus, and ClinicalTrials from January 1, 2000, to July 1, 2026, for studies evaluating gamification and game-based learning in laparoscopic skills education. Eligible studies included interventional and observational studies involving medical students, residents, or surgeons, with measurable performance outcomes. Study quality was assessed using ROBINS-I (Risk of Bias In Non-randomized Studies-of Interventions), RoB 2 (Cochrane Risk of Bias Tool 2), and NIH quality assessment tools. Standardized mean differences (SMDs) were pooled using random-effects models; between-study heterogeneity was quantified by *τ*² and described by the *I*² statistic (inconsistency), while the Cochran Q test tested homogeneity. Certainty of evidence was assessed by the Grading of Recommendations Assessment, Development and Evaluation (GRADE).

**Results:**

A total of 39 studies involving 2102 participants were included. Video game–based interventions served mainly as training tools or assessments, whereas gamified contexts incorporated competitive or collaborative motivational elements. Three subgroup meta-analyses were performed. Game-based learning vs no training yielded a pooled Hedges g of −0.04 (95% CI −7.14 to 7.06; *I*²=96.7%; 95% prediction interval −13.95 to 13.61), game-based learning vs conventional laparoscopic training yielded 0.49 (95% CI −1.39 to 2.36; *I*²=79.4%; prediction interval −3.60 to 4.51), and gamified vs nongamified training yielded 0.87 (95% CI −5.08 to 6.81; *I*²=61.6%). None of the 3 comparisons reached statistical significance, and all prediction intervals crossed the null, indicating that true effects may range from beneficial to harmful across settings. Risk of bias was mostly moderate, and certainty of evidence ranged from very low to moderate. Leave-one-out analysis showed that pooled estimates in the first 2 subgroups were driven largely by individual studies and were not robust.

**Conclusions:**

Both game-based learning and gamified contexts are feasible adjuncts for basic laparoscopic skills training and may enhance motivation and deliberate practice; however, meta-analytic evidence did not demonstrate a statistically significant advantage over no training or conventional laparoscopic training, and the results were not robust to leave-one-out analysis. Generic video games showed limited efficacy for advanced, procedure-specific skills, and the evidence is constrained by heterogeneous outcome measures, small numbers of studies, moderate risk of bias, and a lack of long-term follow-up. As the first synthesis to compare video game-based and gamified context-based approaches within a unified framework, this review provides educators with guidance for integrating task-aligned gamified modules into existing curricula, while highlighting that robust efficacy and sustained operative transfer remain to be demonstrated by larger, standardized studies.

## Introduction

Laparoscopic surgery has become widely adopted across various medical specialties, including general surgery, obstetrics, and urology [1]. Developing effective educational programs to facilitate skill acquisition outside of the clinical setting is crucial for preparing surgeons for proficient surgical performance in the operating room. To date, structured basic skills training programs, such as the Fundamentals of Laparoscopic Surgery (FLS) [2] and Fundamentals of Endoscopic Surgery [3], are widely used. Moreover, cadavers and high-fidelity virtual reality simulators are used for complex laparoscopic skills training, enabling trainees to gain a comprehensive understanding of disease processes, anatomical structures, and procedural steps. 3D reconstruction, 3D printing, and immersive virtual reality technologies, which have been proven effective in surgical planning and skill training, can further strengthen the core visuospatial competencies required for laparoscopic surgery [4-6]. The complexity inherent in laparoscopic skill acquisition involves critical capabilities, including depth perception [7], haptic feedback [8], conversion between 2D and 3D visualization [9], and precise hand-eye coordination [10]. Conventional training methods typically prioritize teaching surgical techniques and promoting trainees’ technical proficiency, but often neglect factors such as fatigue and burnout, which negatively impact learning efficiency and outcomes.

Gamification refers to the implementation of game-design elements within educational contexts to create engaging and self-reinforcing learning experiences and has been widely adopted in fields such as military and aviation training. In medical education, apart from gamified that creates a game-like environment, learning directly developed based on video games is also quite common. The former involves integrating game mechanics to construct a game-like educational context [11], which fosters competition through leaderboards, points, and rewards, or cooperation via friendly interactions and mutual support among trainees. The latter involves the direct application of video games for educational purposes, including entertainment-oriented games and serious games specifically designed for medical skill training, exemplified by Underground (developed by Cutting Edge) [12]. Gamification addresses learners’ needs for context, enhances interpersonal interactions, and stimulates intrinsic motivation [13]. In addition, it can also improve knowledge learning efficiency [14,15] and enhance skills performance across multiple metrics, including task completion time, error rate, and movement economy [16].

Compared with other medical or nonmedical educational contexts, the integration of gamification into laparoscopic training presents unique advantages. Many video game mechanics closely align with key laparoscopic skills [17], suggesting that both fundamental and procedural laparoscopic skills can be effectively enhanced through gamified training approaches. Additionally, performance scores or rankings obtained from video games may serve as supplementary metrics for evaluating laparoscopic competencies, providing educators with novel insights into trainees’ skill acquisition and performance assessment. Previous studies have demonstrated that integrating video games into laparoscopic training significantly improves basic laparoscopic skills [18] and enhances learning efficiency [18]. Gamified contexts have also been shown to enhance laparoscopic skill performance [19] and increase trainees’ motivation for ongoing and subsequent autonomous practice [20,21]. However, a few studies found no significant differences in skill outcomes between video game groups and control groups [22]. The considerable heterogeneity observed in gamification approaches and intervention strategies across existing research indicates ongoing challenges in standardizing the implementation of gamification within laparoscopic training programs.

Several systematic reviews have previously explored gamification in medical education, but their guidance for applying gamified strategies specifically within laparoscopic training remains notably limited. For instance, Xu et al [11] recently reviewed gamified learning in medical education, highlighting its potential to enhance engagement and immersive learning experiences. However, their review primarily addressed nontechnical skills such as clinical reasoning and decision-making. Similarly, earlier reviews by Gupta et al [23] and Jalink et al [24] mainly focused on video game interventions and did not thoroughly address gamified contexts. Gupta et al [23] included only a limited number of laparoscopic-related studies and did not explicitly consider basic laparoscopic skill training, while the review by Jalink et al [24] lacks significant advancements over the past decade. Given the potential value of gamification for laparoscopic skills training and game-based learning and the absence of detailed guidance from existing reviews, a comprehensive systematic evaluation is necessary. This review differs from previously published reviews. It comprehensively and systematically investigated the application of game-related elements in laparoscopic teaching innovatively, identified the current application status of this teaching tool, and provided guidance and references for its future development direction.

This systematic review aims to evaluate the feasibility and effectiveness of gamification and game-based learning in training both basic and advanced skills within laparoscopic education.

## Methods

### Eligibility Criteria

This systematic review was conducted in accordance with the PRISMA (Preferred Reporting Items for Systematic Reviews and Meta-Analyses) guidelines [25]. A protocol was prospectively registered with the PROSPERO database (CRD42024539802) prior to study initiation.

Eligible studies were original research articles investigating gamified interventions for laparoscopic skills training in simulation-based or clinical settings. Gamified interventions were defined as the use of game-like elements to motivate and engage learners in laparoscopic education. Included studies addressed either basic laparoscopic tasks or procedural skills related to laparoscopic surgery, particularly within gastrointestinal, hepatobiliary, or general surgical contexts. Participants were required to have a medical background, including medical students, residents, or practicing physicians. Outcomes were required to have indicators evaluating the laparoscopic skills, including objective metrics like completion time and subjective assessments like Global Operative Assessment of Laparoscopic Skills and FLS.

The exclusion criteria were as follows: (1) non-English publications; (2) full text not available; (3) studies involving veterinary or nonhuman participants; and (4) review articles, conference abstracts, posters, technical notes, and basic science research.

### Search Strategy

We searched 6 databases (PubMed, Embase, Cochrane Library, Scopus, Web of Science, and ClinicalTrials.gov), using the free text words and subject headings to conduct the search strategy. The search time limit was January 1, 2000, to July 1,2026. The full reporting of all literature search components, including database platforms, search syntax, citation tracking, deduplication, and search date, was conducted in accordance with the 16-item PRISMA-S (Preferred Reporting Items for Systematic Reviews and Meta-Analyses literature search extension) checklist [26]. All databases were searched individually rather than via a unified integrated retrieval platform, and all relevant literature was exclusively retrieved from electronic bibliographic databases. Hand searching of reference lists of all included studies was performed to identify additional cited and citing relevant articles for potential inclusion. No attempts were made to contact study authors, clinical experts, device manufacturers, or other personnel to request supplementary unpublished studies, raw data, or additional unpublished results. No published validated search filters (either original or modified versions) were adopted in this review. Besides, no complete or partial search strategies from previously published systematic reviews were adapted or reused for database retrieval. The detailed search strategy and PRISMA-S checklist are presented in Multimedia Appendix 1. Articles included after full-text screening require further reference retrieval. Zotero 6.0.36 (Corporation for Digital Scholarship, Roy Rosenzweig Center for History and New Media at George Mason University) is used to manage all included studies.

### Study Selection

Three authors (XM, ZW, and MS) initially screened all records, excluded clearly irrelevant titles and abstracts, and retrieved the full texts of potentially eligible articles. Two authors (XM and WL) independently reviewed the remaining articles, assessed reference lists, and performed final screening of titles, abstracts, and full texts. Disagreements were resolved through discussion, with a third author (YL) arbitrating when consensus could not be reached.

### Data Collection, Extraction, and Analysis

Data extraction was performed independently by 1 reviewer (XM) using Microsoft Excel. Extracted information included study characteristics (author, year, and design), participant demographics, intervention details, types of gamification approaches, laparoscopic skills, and outcomes. Measurement outcomes included video game rankings and laparoscopic skill performance. For the qualitative synthesis, studies were grouped according to the following four prespecified dimensions: (1) participant population (medical students, surgical residents, or practicing surgeons), (2) intervention type (video game–based training vs gamified instructional contexts), (3) targeted laparoscopic skills (basic tasks such as peg transfer vs advanced procedural skills such as suturing or cholecystectomy), and (4) study design (randomized controlled trials [RCTs], nonrandomized interventional studies, and observational studies). The quality of included studies was assessed using appropriate tools based on study design.

### Pooled Effect

All meta-analyses were performed using R software (version 4.6.1; R Core Team). For all continuous outcomes, the standardized mean difference (SMD) was calculated as the summary effect measure, with Hedges g correction applied to account for small-sample bias. A random-effects model with Hartung-Knapp-Sidik-Jonkman [27] adjustment was applied. Because the included studies assessed laparoscopic performance using different instruments and scales (eg, task completion time in seconds and peg transfer scores), all outcome measures were assumed to assess a common underlying construct of basic or advanced laparoscopic proficiency, thereby justifying the use of SMD to standardize results across heterogeneous metrics. The Nagashima correction [28] was used when calculating prediction intervals

### Heterogeneity

Heterogeneity among studies was quantified using the between-study variance (*τ*²) and described by the *I*² statistic, which expresses the inconsistency of results—the proportion of total variability due to true between-study differences rather than sampling error—as a relative measure, not an absolute measure of the magnitude of heterogeneity. Cochran Q test was used to test the null hypothesis of homogeneity [29].

### Sensitive Analysis

Sensitivity analyses were performed to assess the robustness of the pooled estimates. Given the small number of studies within each subgroup (k=2‐4), formal sensitivity analyses were limited. Where feasible (subgroups with k≥3), a leave-one-out analysis was conducted to examine the influence of individual studies on the pooled effect. For the subgroup comprising only 2 studies (k=2), sensitivity analysis could not be meaningfully performed and results are interpreted with caution.

### Meta-Analysis

In this review, to reduce publication bias, only RCTs with SD and q value were included in meta-analysis, and other studies only adopted qualitative research methods. For studies that only reported median and IQR without SD, we estimated SD using the formula previously described by Wan et al [30]. Studies with severely skewed data and limited sample size were excluded from quantitative synthesis and only narratively summarized.

### Quality Assessment

Included studies were categorized into interventional studies and observational studies. The ROBINS-I (Risk of Bias In Non-randomized Studies-of Interventions) quality assessment tool [31] was used to evaluate the quality of nonrandomized studies and pre-post studies, with ratings classified as low, moderate, high, and no information. RCTs were evaluated using the RoB 2 (Cochrane Risk of Bias Tool 2) quality assessment scale [32], with assessment categorized as low, some concerns, and high. For observational cohort and cross-sectional studies, methodological quality was assessed using the National Institutes of Health (NIH) Quality Assessment Tool for Observational Cohort and Cross-Sectional Studies [33]. The overall quality of each study was graded as good (low risk of bias), fair (moderate risk), or poor (high risk) according to the NIH official criteria. Given the qualitative nature of the synthesis, all studies meeting the eligibility criteria were included in the narrative summary. For the main conclusions, greater interpretive weight was placed on studies with stronger methodological quality (rated as “Good” on NIH tools or “Low risk of bias” on RoB 2 and ROBINS-I), larger sample sizes, and direct relevance to the review question as defined by the inclusion criteria. Studies rated as “Poor” or “High risk of bias” were not excluded but were interpreted with caution, and their influence on the overall conclusions was explicitly noted in the Discussion section.

### Certainty of Evidence

The certainty of evidence for each critical and important outcome was assessed using the GRADE (Grading of Recommendations Assessment, Development and Evaluation) framework [34].

## Results

### Study Characteristics

The initial database search yielded 12,012 records. After title and abstract screening, 93 articles were selected for full-text review. Moreover, 43 records were identified from citation searching, and after removing duplicates, 4 records were selected for full-text review. Following full-text assessment, 39 articles [16,18,20-22,24,35-67] met the inclusion criteria and were included in the final analysis (Figure 1). The included studies consisted of 29 interventional [16,18,20,22,35-37,39,47-67] and 10 observational [21,38-46] studies. The publication years ranged from 2005 to 2026, with the highest number published in 2017 (n=5, 12.82%). Detailed study type of each study is presented in Table 1.

**Figure 1.**
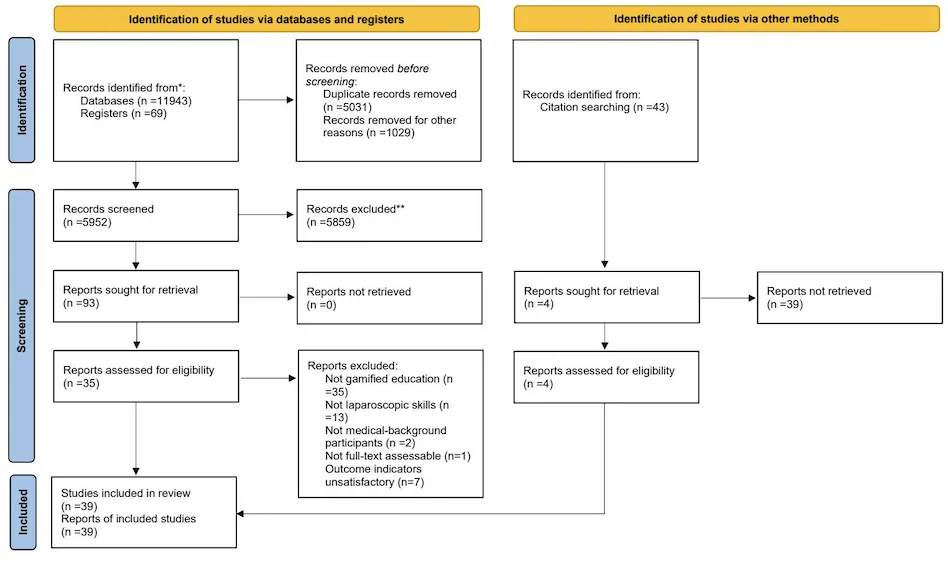
PRISMA (Preferred Reporting Items for Systematic Reviews and Meta-Analyses) flow diagram of search and screening for gamification in laparoscopic education. RCT: randomized controlled trial.

**Table 1.** Summary of participant characteristics, study designs, and theoretical foundations across 39 included studies (2005‐2026).

Items	Values
Surgical specialty, n (%)	
General surgery	179 (52.49)
Obstetrics and gynecology	90 (26.39)
Urology	33 (9.68)
Vascular surgery	10 (2.93)
Endoscopic surgery	10 (2.93)
Otolaryngology	6 (1.76)
Plastic surgery	4 (1.17)
Neurosurgery	4 (1.17)
Cardiac surgery	3 (0.88)
Oral and Maxillofacial Surgery	2 (0.59)
Total	341 (100)
Not reported	1597 (—^a^)
Participant characteristics, n (%)	
Medical students	814 (38.73)
Residents	420 (19.98)
Surgeons	465 (22.12)
Interns	21 (1)
Not specified	355 (16.89)
Total number of participants	2102 (—)
Study types	
RCTs^b^	23 (60)
Non-RCTs	6 (15.4)
Cohort or observational studies	10 (25.6)

a Not applicable.

b RCT: randomized controlled trial.

### Participant Characteristics

A total of 2102 participants were included across all studies. All participants had a medical background, including 814 medical students and 905 physicians (eg, surgeons, residents, and interns; Table 1). Handedness was reported in 723 participants—628 were right-handed, 93 left-handed, and 2 ambidextrous [35]. Participants represented various specialties, including general surgery (n=179), obstetrics and gynecology (n=90), urology (n=33), and vascular surgery (n=10). Moreover, 89 participants reported experience playing musical instruments [16,18,36,37].

### Quality Assessment of the Included Studies

Of the 10 observational studies, 7 were rated fair quality of risk, and 3 good quality. Detailed assessment results are presented in Table 2. Among the 29 interventional studies, 21 were rated as some concerns or moderate risk of bias, 3 were rated as high risk of bias, and 5 were rated as low risk of bias. Detailed assessment results are presented in Figure 2.

**Table 2.** Quality assessment of observational studies (n=10) using the NIH^a^ Quality Assessment Tool for Observational Cohort and Cross-Sectional Studies.

Study	1	2	3	4	5	6	7	8	9	10	11	12	13	14	Overall quality
Datta et al (2020) [38]	Yes	Yes	NR^b^	Yes	Yes	NA^c^	NA	NA	Yes	No	Yes	No	NA	No	Fair
El-Beheiry et al (2017) [21]	Yes	Yes	NR	Yes	No	Yes	Yes	NA	Yes	NA	Yes	No	Yes	No	Fair
IJhosse et al (2020) [39]	Yes	Yes	NR	Yes	Yes	NA	NA	Yes	Yes	No	Yes	No	Yes	Yes	Good
Jalink et al (2014) [40]	Yes	Yes	NR	Yes	Yes	NA	NA	Yes	Yes	No	Yes	No	Yes	Yes	Good
Jalink et al (2015) [41]	Yes	Yes	NR	Yes	No	NA	NA	Yes	Yes	No	Yes	No	Yes	No	Fair
Kulkarni et al (2020) [42]	Yes	Yes	NR	Yes	No	NA	NA	NA	Yes	No	Yes	No	Yes	No	Fair
Overtoom et al (2017) [43]	Yes	Yes	NR	Yes	No	NA	NA	Yes	Yes	No	Yes	No	Yes	No	Fair
Rosser et al (2016) [44]	Yes	Yes	NR	Yes	No	NA	NA	Yes	Yes	No	Yes	No	Yes	No	Fair
Rosser et al (2007) [45]	Yes	Yes	NR	Yes	No	NA	NA	Yes	Yes	No	Yes	No	Yes	No	Fair
Verdaasdonk et al (2009) [46]	Yes	Yes	NR	Yes	No	Yes	Yes	Yes	Yes	Yes	Yes	No	Yes	No	Good

a NIH: National Institutes of Health.

b NR: not reported.

c NA: not applicable.

**Figure 2.**
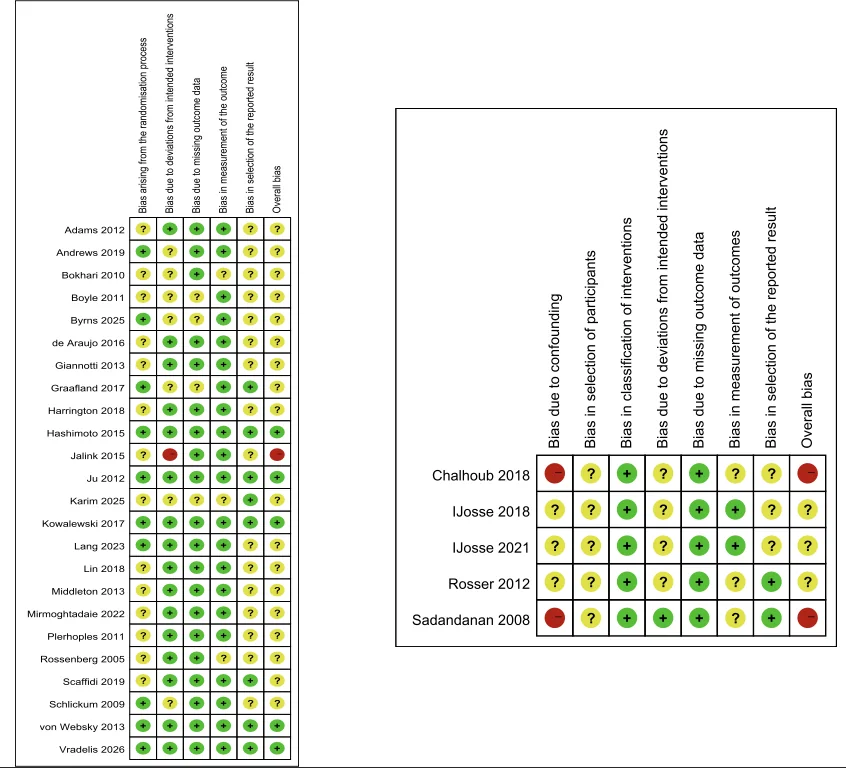
Summary of risk of bias of interventional studies assessed by RoB 2.0 (left) [18,20,22,35-37,47-49,51,53,55,56,58-66] and ROBINS-I (Risk of Bias In Non-randomized Studies-of Interventions; right) [39,50,52,54,57].

### Gamification Approaches of the Included Studies

Gamified strategies in laparoscopic education were categorized into game-based learning and gamified context-based approaches, with 34 articles [18,22,35-45,47-65] and 5 articles [20,21,46,65,66] included, respectively.

Among game-based learning approaches, “Underground” was the most frequently selected game, featured in 9 out of 34 (26.47%) studies [16,39,41,43,44,47-50], necessitating specific Nintendo equipment. The next most popular was “Super Monkey Ball 2,”(developed by SEGA) selected in 6 [44,45,53,55-57] of 34 (17.65%) studies, compatible with Nintendo [44,45,51,52] or intelligent devices [53]. Specific game titles and platforms are listed in Tables 3 and 4 and Textbox 1. The 4 gaming platforms provided are Nintendo, intelligent device, PlayStation, and Xbox.

In the gamified contexts, 4 studies [20,21,46,65] implemented competitive elements (80%), and one study [66] implemented collaborative elements (20%).

**Table 3.** Overview of video games used and laparoscopic skills targeted in included studies (n=3 studies using video game–based approaches).

Single game^a^	Multiple games^b^
Underground [16,38,39,43,45,48,51,52,54]	Boom Blox, Time Crisis 2 [35]
Super Monkey Ball 2 [44,45,53,55-57]	Shooting Range, Pose Mii [37]
Touch Surgery [18,36]	Top Spin, Amped 2, Project Gotham Racing 2 [23]
Table Tennis [37]	Sports Tennis, Table Tennis, Battle at High Altitude [58]
Call of Duty [42,59]	Candy Crush Saga, Temple Run, Crazy Balancing Ball [54]
Silent Scope [45,52]	Whack a Mole, Asteroid Crash, Alien Attacks, Dangerous Route [60]
Star Wars Racer Revenge [45,52]	Trauma Center: New Blood, Resident Evil 4, Need for Speed: Carbon [56]
2D and 3D (custom) [38]	Half Life, Chessmaster [61]
Monkey Ball Banana Blitz [42]	
Dr. Game, Surgeon Trouble [48]	
Marble Mania [62]	
Operation [67]	
Flight Control [63]	

a Single game refers to a video game used by multiple articles or one article.

b Multiple games refer to multiple video games used in the same article.

**Table 4.** Overview of laparoscopic skills targeted in included studies.

Basic skills	Advanced skills
Object transfer [22,23,35,38,40,44,47,50,52,53,57,58,60,64]	Laparoscopic cholecystectomy [20,36,58,65,68]
Cutting [16,38,42,50,51,60]	Inguinal hernia repair [18]
Coordination [16,37,50,54,58,63]	Electrocautery [62]
Suturing [22,35,44,45,52,56]	Searching the small bowel for a Meckel diverticulum [48]
Instrument handling [50,54,58,60]	Performing a biopsy of the parietal peritoneum [48]
Cobra rope drill [45,47,52]	Flap repair surgery [55]
Drop drill [44,45,52]	
Navigation [50,54,58,61,66]	
Clip and grasp [16,37]

Textbox 1. **Overview of theoretical grounding in included studies.**

**Theoretical grounding**

- Skill Transfer Theory [15,22,35,37,45,51,57-60,63]
- Perceptual Learning and Attentional Weighting Theory [38,55,58]
- Deliberate Practice Theory [20,21,46,59]
- Self-Determination Theory [23,46,49,65,66]
- Neuroplasticity Theory [15,38,51]
- Moderating Effect of Cognitive Styles [64]
- Warm-Up Effect Theory [24,52,53,61,62]
- Multimodal Training Theory [18,36,51,64]
- Cognitive Task Analysis Theory [18,36,48]
- Functional Task Alignment Theory [47,50]
- 3D>2D Visual Training Advantage Theory [37,40,42,56,58]
- Adult Learning and Distributed Practice Theory [50,58]
- Feedback Valence Theory [67]

### Feasibility of Gamified Interventions of the Included Studies

Among the included studies, 34 articles applied video games as either training tools or assessment tools for laparoscopic skill development. Out of 34 studies, 26 [16,18,23,35-37,47-64,67] (76.47%) used video games as substitutes for traditional training, with interventions categorized into the following 3 modes: during training, pretraining, and warm-up. The most commonly used mode was during-training (17/26 [16,23,35,37,48,49,51,54-60,64,67], 65.38%). Details are summarized in Table 5. During training refers to the use of self-directed or structured gameplay between 2 tests, pretraining [18,36,50,62] involves completing designated game tasks prior to performance testing, without time constraints, and warm-up [47,52,53,67] refers to short gaming sessions (typically 10‐18 minutes) immediately before testing, without specific game-task requirements.

**Table 5.** Summary of studies using video games as alternative training tools (n=26).

Training phase and author	Year	Training tools	Results
During training			
Harrington et al [16]	2018	Average 5 hours/week voluntarily for 4 consecutive weeks, and completed final video gaming session 24 hours prior to testing.	The intervention group demonstrated significant improvements in dominant (*P*≤.004) and nondominant (*P*<.05) instrument movements, path lengths (*P*≤.04), time taken (*P*≤.02), and end score [*P*≤.046; task-dependent].
Ju et al [35]	2012	Nintendo or PlayStation for 30 minutes between 2 tests.	Participants assigned to Nintendo or PlayStation 2 groups both demonstrated significant improvement in bead transfer and suturing scores. The Nintendo group improved more in bead transfer scores when compared to the PlayStation 2 group (*P*<.05).
Giannotti et al [58]	2013	Nintendo training for 60 minutes a day, 5 days a week, for 4 weeks.	The intervention group showed a significant improvement in performance (*P*<.05) for 13 of the 16 considered performance metrics.
Graafland et al [48]	2017	Regular BLTC^a^, enhanced by 2 separate 30-minute sessions of serious gaming, containing 10 play sessions.	The intervention group solved more problems than the control group (*P*=.03), and recognized a larger proportion of problems, although this parameter was nonsignificant (*P*=.14).
Boyle et al [60]	2011	3 hours on Nintendo.	The intervention group showed a significant performance improvement for one metric in the bead transfer task compared with controls.
Chalhoub et al [54]	2018	Any kind of game.	No significant difference existed in evolution between control and gamers (*P*=.12), nor between intervention and gamers (*P*=.19). Significant advantage was found in evolution of the intervention group over the control group (*P*=.04).
Adams et al [59]	2012	XBOX 360 or Nintendo during 6 weeks.	Residents who played more than 10 hours of video games weekly had the fastest times on the simulator both before and after testing (*P*=.05).
Rosenberg et al [23]	2005	Play any type of game on XBOX over a 2-week period and then play the same game for 2 hours before the second test.	The Top Spin group completed significantly faster in Object Transfer and Tracing (*P*<.05). The Amped 2 group completed faster in Object Transfer, Tracing, and Knot-tying and had better EHC^b^ (in Tracing and Suture Placement). The third Project Gotham Racing 2 group completed faster in Object Transfer and had better EHC in Tracing (*P*<.05).
Middleton et al [37]	2013	Nintendo 1 hour/day for 2 (Group A) or 4 (Group B) consecutive days.	Both groups demonstrated greater improvement, including accuracy in EHC (*P*=.04), faster completion time (*P*=.04), decreased number of left-handed movements (*P*=.03), decreased left-handed total path length (*P*=.03), decreased total number of grasping attempts (*P*=.04), and improved left-handed economy of movement (*P*=.05) for the BCG^c^ task.
Vradelis et al [64]	2026	Complete four 1-hour distributed training sessions (2 sessions before midterm test, 2 after) over 2 weeks.	Serious games are only effective for basic skills and do not significantly improve the overall performance of advanced clamping tasks (*P*=.08); the core advantage of the box trainer is to significantly reduce the error rate of basic operations (*P*<.001).
Byrns et al [51]	2025	After familiarization, each participant performed 6 consecutive, timed attempts of a standardized arthroscopic triangulation task using a suture manipulator, hooks, and rubber bands inside the box trainer.	All groups improved significantly from first to last attempt (*P*<.001): Consultants: time reduced by 44% (103 seconds → 58 seconds), All trainees: time reduced by 39% (161 seconds → 99 seconds); junior trainees: time reduced by 57% (187 seconds → 80 seconds); and medical students: time reduced by 45% (234 seconds → 129 seconds)
de Araunjo et al [55]	2016	All VG groups completed 3 weeks of supervised training on the Nintendo Wii console, with a required minimum of 3 hours of gameplay per week (capped at 7 hours/week to avoid overtraining).	Combined video game groups significantly outperformed the nongaming control (*P*=.003). First-person shooter (ShotG, *P*=.002) and surgery-themed (SurgG, *P*=.02) groups had significantly higher surgical scores versus control; the racing game group showed no significant benefit versus control (*P*=.28), despite the longest training duration
Lin et al [56]	2018	First completed 3 sequential skill-based training games in the video-based box trainer, then finished the subsequent laparoscopic suturing training in the conventional video-based box trainer, with a total training time of 60 minutes	There were no significant differences in baseline characteristics between the 3 groups except for gender distribution, and all 3 training models were well-accepted by participants, with no significant between-group difference in satisfaction and acceptability ratings (all *P*>.05). All participants reported significant improvement in all 5 core domains of laparoscopic suturing skills after training (*P*=.001), with no significant difference in the degree of improvement across the 3 groups.
Mirmoghtadaie et al [49]	2022	The intervention group completed preoperative LC^d^ training via a validated serious game on dedicated tablets, with a mean total game training time of 62.3 (SD 41.1) minutes completed the day before surgery.	The serious game training group significantly outperformed the traditional training group across all measured outcomes: they had a higher preoperative theoretical LC skill score (*P*=.02), required significantly fewer attempts to reach the 80% proficiency threshold (*P*=.001), had a shorter mean operation time (*P*=.03), fewer attempts to complete the operation without complications (*P*=.04), and a higher final surgical skill score (*P*=.01).
Karim et al [67]	2025	Group A received automatic audio-visual negative feedback (buzzer + LED alert for errors); Group B had all feedback disabled. All participants practiced voluntarily for 2 weeks on FLS^e^ peg-transfer tasks.	The negative-feedback group practiced significantly shorter per session (*P*=.03). No significant differences in skill improvement or repeat practice willingness existed between groups. Single negative gamified feedback discouraged sustained practice without boosting laparoscopic performance.
Schlickum et al [61]	2009	40 medical novices were split into 3 arms for 5-week daily systematic game training: Half-Life (3D spatial FPS game), Chessmaster (pure cognitive chess game), and a no-game control group. All took pre- and posttests on MIST-VR and GI Mentor II endoscopic simulators.	Both game groups improved on basic MIST-VR tasks. Only the Half-Life group gained significant progress in GI Mentor navigation tasks. Training with spatially similar video games generated positive skill transfer to endoscopic operation.
Sadandanan et al [57]	2008	30 participants completed baseline laparoscopic tasks (peg transfer, condom cutting, rubber band stretching), then received a 10-minute Super Monkey Ball video game warm-up before repeating the same simulator tests. Paired *t* tests compared pre-post performance.	Posttest scores showed significantly more pegs transferred, fewer drops, faster cutting, and more stretched rubber bands (all *P*<.05). A brief single video game warm-up instantly enhanced basic laparoscopic psychomotor skills, especially for medical students.
Pretraining			
Bokhari et al [62]	2010	Complete 50 levels (randomly repeated 4 times) of the game with the dominant and the nondominant hands (twice each), and then completed the task	The intervention group outperformed the task with fewer errors and superior movement proficiency (*P*<.05), as well as increased ambidexterity with improvement in proficiency of the nondominant hand over time.
IJgosse et al [50]	2018	Train on Underground for 3 sessions and on the FLS video trainer and the LapSim for the fourth session	The Underground group outperformed the control group on Camera Navigation (*P*<.01), Instrument Navigation (*P*<.01), and Coordination (*P*=.01), but no significant effect on Peg Transfer (*P*=.60).
Lang et al [18]	2023	Laparoscopic inguinal hernia modules first on TS^f^ instead of VR^g^	TS group needed significantly fewer attempts to reach proficiency (*P*=.028).
Kowalewski et al [36]	2017	Medical students and surgeons completed LC modules on TS. Medical students of TS group first started LC on TS instead of VR	Surgeons outperformed students in patient preparation (*P*<.001), access and laparoscopy (*P*=.008), LC (*P*<.001), and overall score (*P*=.02).
Warm-up			
Plerhoples et al [53]	2011	10 minutes to warm-up	The intervention group made fewer errors in object positioning (*P*=.01), tissue manipulation task (*P*=.05), and total errors (*P*=.002), but similarly on other metrics.
Jalink et al [47]	2015	15 minutes to warm-up	The intervention group showed a 19.61% improvement in Cobra Rope score, compared to a 0.77% score decrease in group 2 (*P*=.002).
Gentile et al [52]	2012	3 games (6 minutes each) to warm-up	The intervention group showed significantly faster on their first attempt of Cobra Rope drill task. The intervention group had better total suturing scores (*P*<.05).
Andrews et al [63]	2019	28 trainees were randomized: control group directly finished 3 graded LapSim laparoscopic tasks; intervention group warmed up for 10 minutes with PS3 Move motion game Flight Control HD prior to simulation testing.	No performance gaps in easy early tasks. The warm-up group made far more clip errors and simulated blood loss in the hardest vessel-clipping task (*P*<.05). This motion-sensing video game warm-up failed to benefit and even impaired complex laparoscopic performance.

a BLTC: Basic Laparoscopic Technical Course.

b EHC: eye-hand coordination.

c BCG: bimanual clipping and grasping.

d LC: laparoscopic cholecystectomy.

e FLS: Fundamentals of Laparoscopic Surgery.

f TS: Touch Surgery (intelligent device).

g VR: virtual reality.

Among the 34 studies, 9 [22,36-40,42,44,45] (30%) used video game performance or ranking scores as surrogate assessment tools, exploring correlations with laparoscopic skill levels. Detailed outcomes are summarized in Table 6. Kulkarni et al [42] reported strong correlations between scores on XBOX or Nintendo games and performance in laparoscopic tasks, such as Sugar Cube Stacking, Cutting, and total composite scores. IJgosse et al [68] found that participants with greater laparoscopic experience achieved higher scores in the Underground game, although no statistically significant difference was observed between expert and intermediate groups.

**Table 6.** Summary of studies using video game scores as assessment tools for laparoscopic skills (n=9).

Study	Year	Assessment tools	Results
Rosser Jr et al [44]	2017	Game scores from SMB^a^ or Underground	The SMB score had a moderate correlation with intracorporeal suturing (ρ=0.39), and the final score involving all three tasks (ρ=0.39), but low correlations with Pea Drop Drill (ρ=0.11) and FLS Peg Transfer (ρ=0.18). The Underground score had a small correlation with intracorporeal suturing (ρ=0.09) and final score (ρ=0.13).
Datta et al [38]	2020	Game scores from 2D game (customized) and 3D game (customized)	Small significant correlation between performance on the Rope Pass task and the 3D game (Kendall *τ*=-0.151). Small significant correlation between the Paper Cut task and the 2D game (Kendall *τ*=-0.180). Overall laparoscopic performance was also significantly correlated with both the 3D and 2D games (Kendall *τ*=-0.134, Kendall *τ*=−0.163). No significant correlation between the peg transfer task and both games.
Kulkarni et al [42]	2020	Game scores from Call of Duty Operation Black Ops Zombies (XBOX) and Monkey Ball Banana Blitz (Nintendo)	The laparoscopy skills scores correlated significantly with both XBOX and Nintendo scores. There was a stronger correlation between the Nintendo highest score and the total of the laparoscopic skills score (ρ=0.734) compared with the correlation between the XBOX and the total laparoscopic skills score (ρ=.412).
Goris et al [40]	2014	Complete laparoscopy tasks on Nintendo	Game score showed a significant, high correlation with FLS^b^ Peg Transfer (*r*=0.812).
Rosser Jr et al [45]	2007	Game score of Super Monkey Ball 2 (Nintendo), Star Wars Racer Revenge (PlayStation 2), and Silent Scope (XBOX)	All video games used to quantify the subjects’ demonstrated video game skill were highly correlated with laparoscopic skill and suturing ability. Super Monkey Ball 2 showed the highest correlation (*r*=0.63), followed by Silent Scope (*r*=0.50).
IJgosse et al [39]	2020	Game scores from Underground (Nintendo)	Both experts (*P*=.02) and intermediates (*P*=.04) outperformed novices in terms of task completion speed, but experts did not outperform intermediates. No correlations were calculated.
Rosenberg et al [22]	2005	Scores of Top Spin, Street Race, Cone Challenge, and Amped 2.	The laparoscopic task measurement that best correlated with video game performance was time to complete the objective. This difference was more pronounced in the simpler tasks (object transfer and figure-of-eight). The more-complex tasks (suturing and knot-tying) showed little difference.
Kowalewski et al [36]	2017	Scores of patient preparation, access and laparoscopy, and LC from Touch Surgery	Surgeons outperformed students in all 3 tasks. No correlations were calculated.
Middleton et al [37]	2013	Nintendo Wii Scores	Better game score rankings demonstrated a moderate correlation with decreased time to completion (ρ=0.5), faster right-handed speed (ρ=0.694), and decreased right-handed total path length (ρ=0.521) for the EHC^c^ task. No significant correlations were found for the BCG^d^ and the THM^e^ tasks.

a SMB: Super Monkey Ball.

b FLS: Fundamentals of Laparoscopic Surgery.

c EHC: eye-hand coordination.

d BCG: bimanual clipping and grasping.

e THM: two-handed manipulation.

Creating a competitive context mainly involves rewarding top-ranked participants and regularly updating the leaderboard. Websky et al [20] developed a peer-group benchmarks group to enhance collaborative settings, which resulted in better performance in efficiency and training motivation relative to the control group. Specific interventions and results are listed in Tables 7 and 8.

**Table 7.** Summary of included studies on gamified contexts implementation.

Author	Year	Gamified contexts	Results
Verdaasdonk et al [46]	2009	An online competition	More attempts (>30, *P*=.008) correlated with higher scores. The motivation score did nonsignificantly correlate with the final score on the simulator.
El-Beheiry et al [21]	2017	Leaderboards are advertised both in class and via email on a biweekly (top 3) and monthly basis (top 10)	The mean simulator usage time was significantly higher in CC^a^ in the fourth month of observation (*P*=.011), and the opposite was found in the fifth month (*P*=.007). CC finished the course earlier (*P*=.01), and were significantly faster (*P*=.03) in Peg Transfer than the control group. More residents in CC continued to use the simulator voluntarily after the course (*P*=.002).
Hashimoto et al [65]	2015	The top performer after 10 sessions would be awarded a gift card for a flight simulator experience (valued at approximately $150)	During the first LC^b^, CC performed better in completion time (*P*=.01), movements (*P*=.04), and path length (*P*=.04). During the final LC, CC performed better in movements (*P*=.02) and path length (*P*=.02). CC reported higher mean state anxiety before (*P*<.001) and after (*P*<.001) the study than the control group.
Websky et al [20]	2013	Peer-group benchmarks group (PGDG)	PGDG performed significantly better in completion time and right-hand instruments path length (both *P*<.001) than the control group. PGDG had more visits (*P*<.001).
Satchwell et al [66]	2019	A colon-themed progressive game narrative, real-time performance leaderboard, achievement badges, and hourly top-performer rewards, and a final grand prize for overall top rank	The gamified curriculum group showed significantly superior clinical performance in live patient colonoscopies, the study’s primary endpoint. Blinded expert assessment using the validated JAG DOPS^c^ scale found a highly significant between-group difference in overall technical skill (*P*=.001), with the gamified group also achieving a significantly higher cecal intubation rate (88.9% vs 61.1%, *P*=.047) and shorter procedure time for successful intubations (*P*=.002).

a CC: competitive cohort.

b LC: laparoscopic cholecystectomy.

c JAG DOPS: JAG Direct Observation of Procedural Skills.

**Table 8.** Summary of included studies on training tools and gamification elements.

Author	Type	Training tool	Gamification elements
Verdaasdonk et al [46]	Competitive	Laparoscopic VR^a^ simulator	Multicenter online skills competition, real-time online leaderboard and public score display, quantitative scoring system, material reward, and unlimited practice attempts
El-Beheiry et al [21]	Competitive	VR or simulation-based laparoscopic modules (unspecified platform)	Skills competition, leaderboard, prizes, and bragging rights
Hashimoto et al [66]	Competitive	Laparoscopic simulator (possibly VR-based)	Real-time feedback, task scoring, and staged challenges
von Websky et al [20]	Mixed	VR Endoscopy simulator	Leaderboard, badges, progress bars, and staged challenges
Satchwell et al [66]	Collaborative	Simulation-based modules	Learners earn points, unlock badges, and advance storylines; tasks completed in teams to get group rewards

a VR: virtual reality.

### Meta-Analysis

Of all 39 studies included in this review, 9 studies [20,35,48,49,51,56,58,63,65] matched the criteria for inclusion in the meta-analysis. Of the total, 3 [51,58,63] studies compared game-based learning with a no-intervention (blank) control group, 3 [48,49,56] compared it with conventional laparoscopic training, and 2 [20,65] examined gamified vs nongamified learning. Only 1 study [35] compared different video game platforms.

Subgroup meta-analyses were conducted to compare the effectiveness of game-based learning against different control conditions on laparoscopic skill performance. The SMD was used as the effect measure, and data were pooled with a random-effects model. Furthermore, 4 studies [20,48,58,65] required the use of the formula by Wan et al [30] to estimate the SD from IQR. The detailed data were shown in Multimedia Appendix 2.

As illustrated in Figure 3, 3 studies [51,58,63] (95 participants) evaluated game-based learning vs no training. The pooled Hedges *g* was –0.04 (95% CI –7.14 to 7.06), with a 95% prediction interval ranging from −13.95 to 13.61. The *I*² statistic indicated noteworthy inconsistency (*I*²=96.7%), with a between-study variance of *τ*²=7.89 (Q=59.88; *P*<.001).

**Figure 3.**
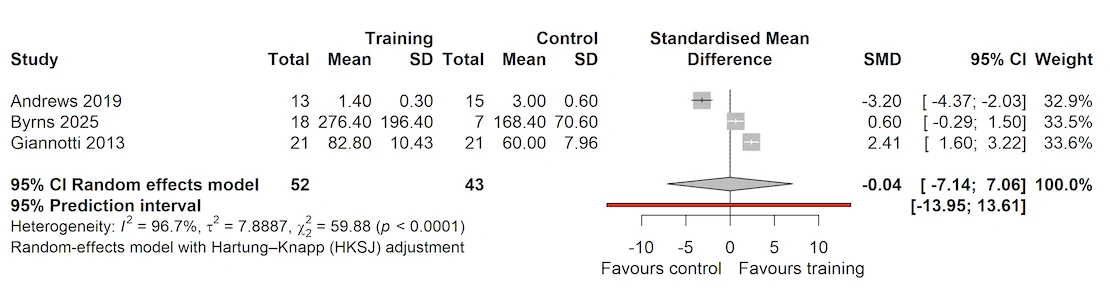
The effect of game-based learning compared with no training showed a better outcome than the no-training group [51,58,63]. SMD: standardized mean difference.

As illustrated in Figure 4, 3 studies [48,49,56] (108 participants) compared game-based learning with conventional laparoscopic training. The pooled Hedges *g* was 0.49 (95% CI –1.39 to 2.36), with a 95% prediction interval ranging from –3.60 to 4.51. The *I*² statistic indicated noteworthy inconsistency (*I*²=79.4%), with a between-study variance of *τ*²=0.45 (Q=9.69; *P*=.008).

**Figure 4.**
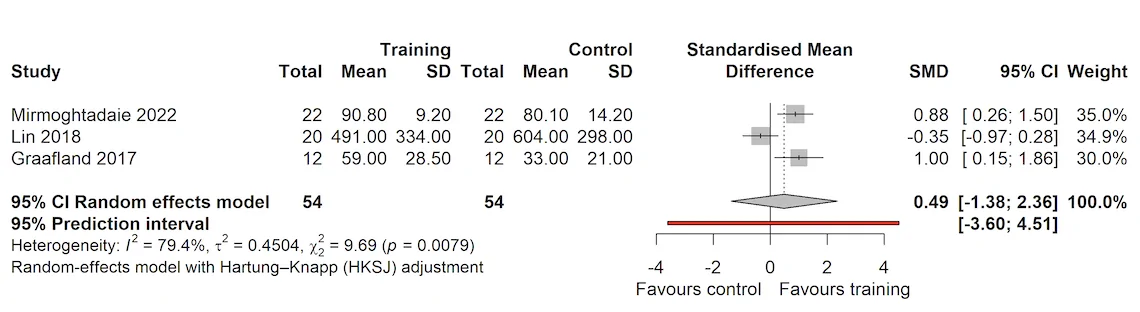
Game-based learning tended to be more effective than conventional laparoscopic training, with the exception of the study by Lin et al [48,49,56]. SMD: standardized mean difference.

As illustrated in Figure 5, 2 studies [20,65] (86 participants) compared gamification training with nongamification training. The current outcome was task completion time, where smaller values correspond to better performance. Raw time data were negated before calculating Hedges *g* to align effect directions with other end points. A positive effect size thus indicates better performance among participants receiving training. Under the random-effects model, the pooled Hedges *g* was 0.87 (95% CI –5.08 to 6.81). The *I*² statistic indicated inconsistency (*I*²=61.6%), with a between-study variance of *τ*²=0.29 (Q=2.61; *P*=.11).

**Figure 5.**
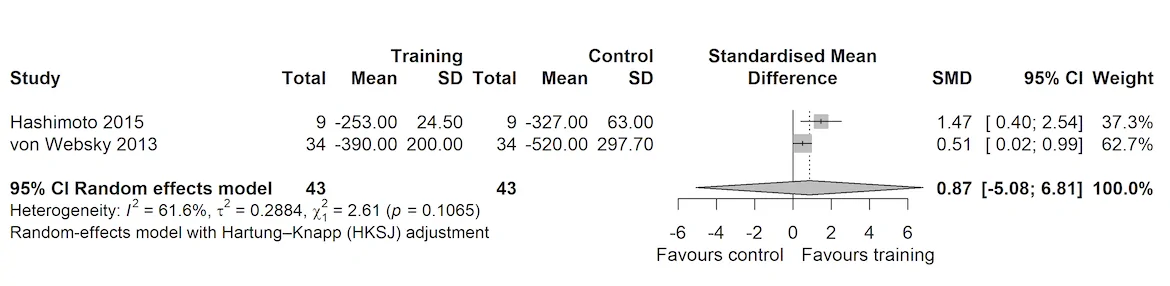
Gamification showed a significant improvement over nongamified learning [20,65]. SMD: standardized mean difference.

A study by Ju et al [35] showed a further comparison between different video games. Both groups achieved statistically significant improvements in the bead transfer task (Wii: mean score improvement 62, SD 14; *P*<.001; PS2: mean score improvement 40, SD 14; *P*=.01), yet the between-group difference in improvement magnitude did not reach statistical significance. For the intracorporeal suturing task, neither group showed a statistically significant change in performance after the intervention. Double counting in the meta-analyses did not occur.

### Sensitivity Analysis

To determine whether the pooled estimate was driven by a single study, we performed leave-one-out sensitivity analyses (Figure 6). The SMD reversed direction after omitting one of the following studies: (1) study by Andrews et al [63]: 1.52 (95% CI –9.96 to 13.00), (2) study by Byrns et al [51]: –0.38 (95% CI –36.04 to 35.28), (3) study by Giannotti et al [58]: –1.28 (95% CI –25.46 to 22.90), and every 95% CI still spanned the null, indicating a nonrobust pooled estimate.

**Figure 6.**
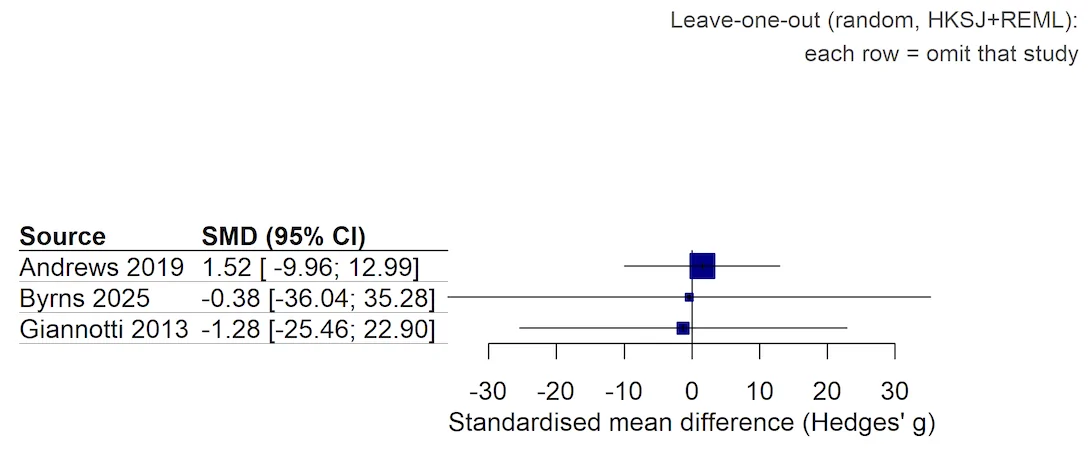
Leave-one-out sensitivity analysis for subgroup 1 [51,58,63]. HKSJ: Hartung-Knapp-Sidik-Jonkman; SMD: standardized mean difference.

The pooled estimate for subgroup 2 was highly sensitive to the included studies (Figure 7). The overall nonsignificant result for group 1 was attributable mainly to the study by Lin et al [56]: after its exclusion, the pooled SMD shifted to 0.92 (95% CI 0.17 to 1.67; *P*=.04) with *I*²=0%, whereas excluding the study by Mirmoghtadaie et al [49] or Graafland et al [48] did not change the nonsignificant conclusion (SMD 0.29, 95% CI –8.29 to 8.88; and SMD 0.27, 95% CI –7.54 to 8.07, respectively).

**Figure 7.**
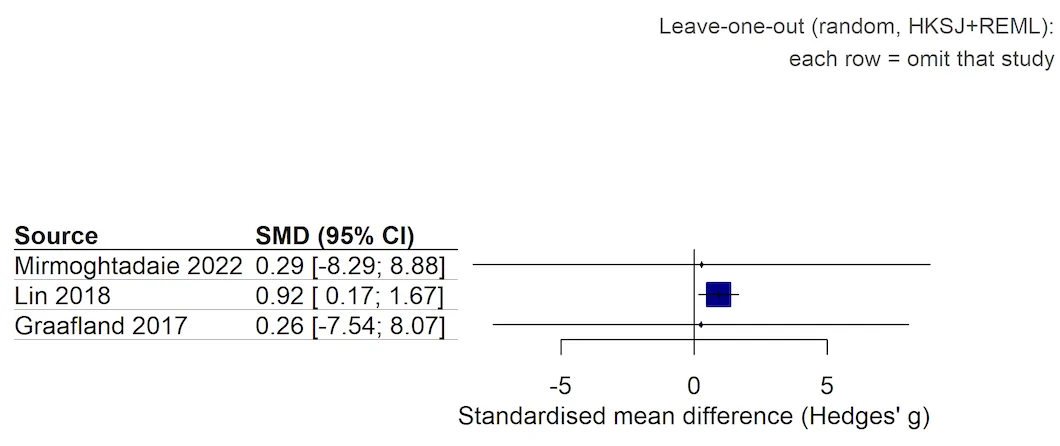
Leave-one-out sensitivity analysis for subgroup 2 (Mirmoghtadaie et al [49], 2022; Lin et al [56], 2018; and Graafland et al [48], 2017). HKSJ: Hartung-Knapp-Sidik-Jonkman; SMD: standardized mean difference.

### Certainty of Evidence

GRADE certainty assessment was performed for three comparisons of laparoscopic training interventions. Evidence for gamification versus nongamification was moderate certainty. Evidence for game-based learning vs no training was low certainty. Evidence for game-based learning versus conventional laparoscopic training was very low certainty. Detailed GRADE assessments are provided in Multimedia Appendix 3.

## Discussion

### Principal Findings

This systematic review evaluated the feasibility and effectiveness of gamified interventions, including game-based learning and gamified context-based approaches, in laparoscopic skills training. Video games were primarily used as supplementary training tools or assessment platforms, whereas gamified contexts incorporated motivational elements, such as competition and collaboration, to enhance learner engagement and participation [16,39,45,49,69,70]. Overall, qualitative evidence suggested that gamified interventions may provide educational benefits in laparoscopic training, particularly in improving psychomotor performance, cognitive outcomes, and learner motivation [16,20,21,49,58,65]. However, the magnitude of these effects appeared to vary depending on the training objectives, intervention characteristics, and assessment domains.

Previous studies have reported that video game-based training may improve basic psychomotor abilities, including task completion time, movement efficiency, and error reduction among surgical trainees [35,37,59,62]. Serious games, such as Touch Surgery (developed by Kinosis Ltd) and Dr. Game, Surgeon Trouble (developed by Amsterdam UMC), have also shown potential value in supporting theoretical knowledge acquisition, procedural understanding, and intraoperative decision-making [18,36,48]. Nevertheless, the transferability of these benefits to advanced laparoscopic performance remains uncertain. Current evidence suggests that video games may facilitate basic visuospatial and psychomotor skill development but have limited ability to reproduce the procedural complexity, instrument manipulation, and cognitive demands required for advanced operative tasks [22,50,64].

Compared with conventional laparoscopic training, game-based learning demonstrated comparable performance outcomes in selected basic skill tasks but has not consistently demonstrated superiority for procedure-specific training [56]. This may be related to differences in intervention design, insufficient surgical specificity, variable training exposure, and heterogeneous outcome assessment methods across studies [16,42]. Therefore, video games are more appropriately considered complementary training resources rather than replacements for established simulation-based curricula.

Gamification strategies may provide additional educational value by modifying learner motivation and engagement through structured feedback, competition, or collaboration [23,46,65,66]. Competitive gamification has been associated with improvements in dexterity, performance efficiency, and practice motivation. For example, Hashimoto et al [65] reported reduced movement variability and shorter instrument paths following competitive training, while El-Beheiry et al [21] observed improved task completion and increased voluntary practice in competitive environments. In contrast, collaborative gamification may facilitate teamwork, communication, and sustained learner participation, suggesting that different gamification elements may influence distinct aspects of surgical education [20,54].

Despite these encouraging findings, the current evidence remains limited by considerable variation in intervention protocols, target skills, participant characteristics, and outcome measurements [16,45]. Although gamified approaches are accessible and potentially cost-effective compared with traditional simulation platforms, many commercial video games lack sufficient surgical specificity and may not adequately reproduce the technical and cognitive demands of laparoscopic procedures [16,45]. Further well-designed studies with standardized training protocols, validated assessment tools, and clinically relevant outcomes are required to determine the optimal role of gamification in surgical education.

Pooled effects were small to moderate and none reached statistical significance. Inconsistency among studies was substantial (*I*²=61.6%‐96.7%); because *I*² measures inconsistency rather than the magnitude of heterogeneity, between-study dispersion is better reflected by the between-study variance (τ²) and, above all, by the prediction intervals, which were far wider than the CIs and crossed the null in every comparison. The true effect may therefore range from harmful to beneficial across settings, and a uniform benefit of gamification cannot be assumed. It is important to distinguish the average effect from the distribution of true effects. Although the three pooled estimates were small (near-zero for game-based learning versus no training, or moderate, none was statistically significant, and the 95% prediction intervals were far wider than the CIs and crossed the null in every comparison. This indicates that, beyond the average effect, the true effect in a given setting is likely to vary widely—from negligible or harmful to substantial benefit, so the benefit of gamification cannot be assumed to be uniform across populations, training protocols, or outcome domains. Notably, the very wide prediction intervals may reflect not only genuine between-study dispersion but also the small number of studies in each subgroup, since with so few studies the between-study variance (*τ*²) is estimated imprecisely and the intervals become correspondingly uncertain. Reporting only *I*² would have obscured this, because a high *I*² is a proportion and indicates neither the magnitude nor the clinical relevance of the dispersion.

### Heterogeneity

The *I*² statistic indicated substantial inconsistency across all 3 subgroups. Differences in outcome types may represent the most important potential contributor. Such heterogeneity was expected given the diversity of training interventions, assessment methods, and participant characteristics included in this review. The outcomes included distinct dimensions of laparoscopic performance, including time efficiency, accuracy, error rates, and technical skills. Laparoscopic performance is a multidimensional construct encompassing psychomotor coordination, efficiency, accuracy, and cognitive decision-making. Therefore, combining these outcomes into a single estimate may obscure domain-specific effects and contribute to substantial heterogeneity.

The pooled studies also differed in what was measured. This included outcomes from different performance domains (time-based efficiency, error counts, kinematic measures such as path length and movements, technical scores, problem-solving and situational awareness, and nontechnical measures such as state anxiety [16,20,37,48,59,65]), tasks at different levels (basic transfer and suturing [22,56,58] vs cholecystectomy and other procedural tasks [20,49,65]), and inconsistent outcome direction, since “lower is better” measures (time, errors, and path length) required reversal before pooling. Pooling such heterogeneous outcome constructs into a single standardized mean difference therefore limits the interpretability of the pooled estimate and likely contributes to the high between-study dispersion observed. In addition, training duration and training modalities were also important potential sources of variability. Longer training durations tended to be associated with greater performance improvements, suggesting a possible dose-response relationship between training exposure and skill acquisition. In contrast, video games used solely as warm-up activities were unlikely to produce significant improvements and might even have limited benefits for physical skill development.

### Certainty of Evidence and Robustness of Findings

The robustness of the pooled estimates was examined using leave-one-out sensitivity analysis. This revealed that the pooled estimate for the game-based learning vs no-training subgroup was not robust; the SMD reversed direction and every 95% CI still spanned the null after omitting any single study (Andrews and Hewett [63], Byrns et al [51], or Giannotti et al [58]). In the game-based learning vs conventional training subgroup, the overall nonsignificant result was driven largely by a single study; its exclusion shifted the pooled estimate to a statistically significant positive value and eliminated inconsistency, whereas excluding the other 2 studies did not change the nonsignificant conclusion. Given the small number of studies in each subgroup (k=2‐4) and the substantial between-study heterogeneity observed, these results should be interpreted with caution and are not sufficient to establish robust effectiveness.

According to the GRADE framework, the certainty of evidence was rated as moderate to very low across the evaluated outcomes. The main reasons for downgrading were substantial inconsistency among studies, methodological limitations, limited sample sizes, and imprecision of effect estimates. Although gamified interventions demonstrated potential benefits in laparoscopic training, the current evidence remains insufficient to support definitive conclusions regarding their superiority over conventional training approaches.

To translate gamification and game-based learning into meaningful educational practice, careful instructional design is essential. Instead of using commercial video games in isolation, future efforts should prioritize the development or customization of task-aligned, evidence-informed gamified modules that complement existing laparoscopic curricula [44,59]. Embedding competitive or collaborative game elements within simulation-based training can promote learner autonomy, increase deliberate practice, and enhance motivation [21,65]. However, the design must avoid overemphasizing entertainment at the expense of clinical relevance or technical fidelity [50]. Alignment with validated assessment tools, such as OSATS (Objective Structured Assessment of Technical Skills) [71] or FLS metrics, can enhance educational value while ensuring objective evaluation [35,48,55,65]. Instructors should also consider learner variability when integrating gamified approaches—tailoring difficulty levels, feedback frequency, and reward mechanisms to the learner’s stage of training may optimize both engagement and skill acquisition. As interest grows in intelligent surgical training ecosystems, gamification could play a complementary role when combined with adaptive learning algorithms and real-time performance analytics [38,55,58].

### Limitations

Despite the promising findings, this review has several limitations. First, substantial heterogeneity existed in gamification formats, training duration, participant characteristics, assessment tools, and outcome measures, which limited the comparability among studies and complicated quantitative synthesis. Second, several potentially eligible studies could not be included in the meta-analysis because they reported medians and IQRs rather than means and SDs. Although statistical conversion methods are available, such transformations may introduce estimation uncertainty and potential bias. Consequently, subgroup analyses were restricted by the limited number of available studies. Third, the small number of studies in each subgroup (k=2‐4) was below the minimum threshold (typically ≥10) recommended for testing funnel plot asymmetry, so small-study effects could not be reliably assessed. Notably, such tests detect small-study effects—a pattern in which smaller studies report systematically larger effects—which may arise from publication bias but also from other causes such as poorer methodological quality in smaller studies, true heterogeneity, or chance. Because publication bias and other contributors to asymmetry cannot be excluded, pooled estimates should be interpreted cautiously. Fourth, although both interventional and observational studies were included, several studies had methodological limitations, particularly regarding randomization procedures, allocation concealment, and blinding, which may have affected the certainty of evidence. Fifth, most studies evaluated short-term skill acquisition rather than long-term retention or clinical transfer. Although gamification may enhance motivation and engagement during initial training, whether these benefits can be sustained over prolonged training periods remains unclear.

Future studies should adopt standardized outcome measures, clearly define training protocols, and incorporate longitudinal follow-up to evaluate long-term educational effects. Researchers should also report complete statistical parameters, including means and SDs, to facilitate future evidence synthesis. Furthermore, future investigations should explore whether adaptive gamification strategies, such as dynamic difficulty adjustment, personalized feedback, and progressive challenges, can maintain learner engagement and support sustained skill development throughout extended training programs.

### Conclusion

In summary, this review confirms that gamified interventions and game-based learning are feasible adjuncts for laparoscopic skills education and may support basic psychomotor skill acquisition and learner motivation; however, the pooled meta-analytic estimates did not reach statistical significance and were not robust to leave-one-out analysis, so a definitive advantage over conventional training cannot be established. Game-based learning and gamified contexts each have distinct strengths; the former serves well as supplementary training or warm-up tools, while the latter offers a framework for curriculum-wide engagement design. A key finding is that generic video games do not reliably improve advanced procedural competence, underscoring the need for task-aligned, curriculum-integrated gamified modules rather than isolated commercial games. This review differs from prior work by comparing both major gamification approaches within a single analytical framework and by mapping their underlying theoretical foundations to the evidence base. Its contribution lies in providing educators with an evidence-informed rationale for selecting specific gamified strategies based on training objectives—competitive elements for technical proficiency, collaborative elements for team-based skills—and in highlighting implementation realities, including the motivation paradox whereby positive attitudes do not guarantee sustained voluntary practice. Looking ahead, the integration of gamified instructional design with adaptive learning algorithms and standardized assessment tools may represent the next step toward personalized, proficiency-based laparoscopic training. Until then, gamification should be viewed as a valuable complement to, rather than a replacement for established simulation methods.

## Supplementary material

10.2196/81331 Checklist 1 PRISMA_2020_expanded_checklist.

10.2196/81331 Checklist 2 PRISMA_2020_abstract_checklist.

10.2196/81331 Multimedia Appendix 1 Searching strategy.

10.2196/81331 Multimedia Appendix 2 Data_extraction_Meta_2026.

10.2196/81331 Multimedia Appendix 3 Should game-based learning and gamification vs other types be used for laparoscopic education.

## Abbreviations

FLS: Fundamentals of Laparoscopic Surgery
GRADE: Grading of Recommendations Assessment, Development and Evaluation
NIH: National Institutes of Health
OSATS: Objective Structured Assessment of Technical Skills
PRISMA: Preferred Reporting Items for Systematic Reviews and Meta-Analyses
PRISMA-S: Preferred Reporting Items for Systematic Reviews and Meta-Analyses literature search extension
RCT: randomized controlled trial
RoB 2: Cochrane Risk of Bias Tool 2
ROBINS-I: Risk of Bias In Non-randomized Studies-of Interventions
SMD: standardized mean difference
