# Supplementary material for: Game-Based Learning and Gamification in Laparoscopic Skills Education: Meta-Analysis and Systematic Review
Source: JMIR Serious Games. 2026 Oct 29;14:e81331. doi: 10.2196/81331 (PMC13718743; doi:10.2196/81331)
Supplement: Supplementary file 2 [file games-v14-e81331-s004.pdf]

Authors, Year	Outcome type	experimental group	Mean ± SD (n)	Controlled group	Mean ± SD (n)	p value
Byrns et al 2025[40]	Post-training completion time of peg transfer	Lap Box	168.4 ± 70.6(18)	No train	276.4 ± 196.4(7）	<0.001
Bokhari et al 2010[47]	Electrocoagulation task time (standardized score)	Wii	0.25 ± 0.07(14)	No train	0.63±0.1(7)	<0.05
Andrews et al 2019	Total clip errors	PS3	3.0±0.6(13)	No train	1.4±0.3(15)	0.02
Giannotti et al 2013[33]	Accuracy rate of eye-hand coordination	Wii	82.8 ± 10.43(21)	No train	60.0 ± 7.96(21)	<0.0001
Ju et al 2012[29]	Variation of bead transfer score	Wii	62±14(22)	PS2(Time Crisis 2)	40±14(20)	<0.001
Mirmoghtadaie et al 2022[22]	Surgery skill score	Serious game(Touch Surgery)	90.8±9.2(22)	pattern training	80.1 ± 14.2(22）	0.012
Lin et al 2018[42]	Suturing time (s)	Video game	604 ± 298（20）	Video box trainer	491 ± 334（20）	0.322
Graafland et al 2017[36]	Problem resolution ratio	Game-enhanced curriculum	59% ± 28.5%(12)	Regular BLTC	33% ± 21.0%(12)	0.03
El-Beheiry 2017	completion time of peg transfer	Ranking list	194±66 (25)	Regular courses	233±53 (24)	0.03
Hashimoto 2015	Final LC motion times	Competitive Training (Ranking List + Prizes)	253±24.5(9)	Regular courses	327±63.0(9)	0.02
von Websky 2013	Time of gallbladder removal (s)	PGD	390 ± 200.0(34)	SC	520 ± 297.7(34)	<0.001
