# Supplementary material for: Game-Based Learning and Gamification in Laparoscopic Skills Education: Meta-Analysis and Systematic Review
Source: JMIR Serious Games. 2026 Oct 29;14:e81331. doi: 10.2196/81331 (PMC13718743; doi:10.2196/81331)
Supplement: Supplementary file 3 [file games-v14-e81331-s005.pdf]

Author(s):
Question: Game-based learning and gamification compared to other types for laparoscopic education
Setting:
Bibliography:

Certainty assessment							№ of patients		Effect		Certainty	Importance
№ of studies	Study design	Risk of bias	Inconsistency	Indirectness	Imprecision	Other considerations	game-based learning and gamification	other types	Relative (95% CI)	Absolute (95% CI)		
**New outcome**												
4	randomised trials	serious[a]	not serious	serious[b]	not serious	publication bias strongly suspected all plausible residual confounding would reduce the demonstrated effect[c]	66/116 (56.9%)	50/116 (43.1%)	-- (-0.31 to 3.47)	**-- per 1,000** (from -- to --)	⨁⨁◯◯ Low[a,b,c]	
**New outcome**												
3	randomised trials	serious[a]	serious[d]	serious[b]	not serious	publication bias strongly suspected dose response gradient[c]	54/108 (50.0%)	54/108 (50.0%)	-- (-0.14 to 1.32)	**-- per 1,000** (from -- to --)	⨁◯◯◯ Very low[a,b,c,d]	
**New outcome**												
2	randomised trials	not serious	not serious	serious[b]	not serious	publication bias strongly suspected dose response gradient[c]	43/86 (50.0%)	43/86 (50.0%)	-- (-0.12 to 1.48)	**-- per 1,000** (from -- to --)	⨁⨁⨁◯ Moderate[b,c]	

CI: confidence interval

Explanations

a. use ROB 2.0 tool to assess the risk of bias, 3 studies were all confirmed as some concerns
b. the types of outcomes are various and hard to describe the overall effect
c. small sample size makes it hard to put publication bias
d. Lin et al's study showed a contrary outcome to other studies
