# Supplementary material for: Game-Based Learning and Gamification in Laparoscopic Skills Education: Meta-Analysis and Systematic Review
Source: JMIR Serious Games. 2026 Oct 29;14:e81331. doi: 10.2196/81331 (PMC13718743; doi:10.2196/81331)
Supplement: Supplementary file 5 [file games-v14-e81331-s003.docx]

PubMed：

(surgeon*[tiab] OR resident*[tiab] OR trainee*[tiab] OR "medical student*"[tiab] OR "surgical trainee*"[tiab] OR dexterity[tiab]) AND (gamification[mh] OR "video game*"[tiab] OR "virtual reality"[mh] OR "augmented reality"[tiab] OR "computer simulation"[mh] OR "serious game*"[tiab] OR "game-based"[tiab] OR "head-mounted"[tiab] OR simulator*[tiab]) AND (laparoscopy[mh] OR "minimally invasive surgical procedures"[mh] OR laparoscop*[tiab] OR "minimally invasive"[tiab]) AND ("education, medical"[mh] OR "clinical competence"[mh] OR "simulation training"[mh] OR "education, medical, graduate"[mh] OR "internship and residency"[mh] OR educat*[tiab] OR train*[tiab] OR "technical skill*"[tiab] OR psychomotor[tiab])

n=2857

Cochrane:

#1	MeSH descriptor: [Gamification] explode all trees	37

#2	MeSH descriptor: [Video Games] explode all trees	1550

#3	MeSH descriptor: [Virtual Reality] explode all trees	1972

#4	MeSH descriptor: [Simulation Training] explode all trees	2128

#5	MeSH descriptor: [Computer Simulation] explode all trees	5106

#6	((gamificat*) OR "game-based learning" OR "game based learning" OR (serious NEXT gam*) OR (educational NEXT gam*) OR (video NEXT gam*) OR "virtual reality" OR "augmented reality" OR "immersive"):ti,ab,kw	15589

#7	#1 OR #2 OR #3 OR #4 OR #5 OR #6	20017

#8	MeSH descriptor: [Laparoscopy] explode all trees	9853

#9	MeSH descriptor: [Minimally Invasive Surgical Procedures] explode all trees	47181

#10	(laparoscop* OR "minimally invasive" OR "minimally invasive surgery" OR keyhole):ti,ab,kw	43281

#11	#8 OR #9 OR #10	78932

#12	MeSH descriptor: [Education, Medical] explode all trees	5186

#13	MeSH descriptor: [Clinical Competence] explode all trees	6026

#14	MeSH descriptor: [Simulation Training] explode all trees	2128

#15	MeSH descriptor: [Internship and Residency] explode all trees	2133

#16	MeSH descriptor: [Internship and Residency] explode all trees	2133

#17	(surgeon* OR resident* OR trainee* OR (medical NEXT student*) OR (surgical NEXT trainee*) OR "surgical education" OR "postgraduate training" OR skill* OR train* OR educat* OR psychomotor OR "hand-eye" OR dexterity):ti,ab,kw	356881

#18	#12 OR #13 OR #14 OR #15 OR #16 OR #17	357505

#19	#7 AND #11 AND #18	883

Embase:

('gamification'/exp OR 'video game'/exp OR 'virtual reality'/exp OR 'computer simulation'/exp OR gamificat*:ti,ab,kw,de OR 'game-based':ti,ab,kw,de OR 'serious gam*':ti,ab,kw,de OR 'video gam*':ti,ab,kw,de OR 'computer gam*':ti,ab,kw,de OR 'virtual reality':ti,ab,kw,de OR 'augmented reality':ti,ab,kw,de OR 'mixed reality':ti,ab,kw,de OR 'head mounted':ti,ab,kw,de) AND ('laparoscopy'/exp OR 'minimally invasive surgery'/exp OR laparoscop*:ti,ab,kw,de OR 'minimally invasive':ti,ab,kw,de OR 'minimally invasive surgery':ti,ab,kw,de OR 'keyhole':ti,ab,kw,de) AND ('medical education'/exp OR 'clinical competence'/exp OR 'simulation training'/exp OR surgeon*:ti,ab,kw,de OR resident*:ti,ab,kw,de OR trainee*:ti,ab,kw,de OR 'medical student*':ti,ab,kw,de OR 'surgical trainee*':ti,ab,kw,de OR 'surgical education':ti,ab,kw,de OR 'postgraduate training':ti,ab,kw,de OR skill*:ti,ab,kw,de OR train*:ti,ab,kw,de OR educat*:ti,ab,kw,de OR psychomotor:ti,ab,kw,de OR 'hand-eye':ti,ab,kw,de OR dexterity:ti,ab,kw,de)

n=3608

Scopus:

TITLE-ABS-KEY("game-based learning" OR "game based learning" OR gamifica* OR "serious gam*" OR "educational gam*" OR "video gam*" OR "computer gam*" OR "simulation gam*" OR "virtual reality" OR "augmented reality" OR "mixed reality" OR immersive OR "head mounted") AND TITLE-ABS-KEY(laparoscop* OR laparoscopy OR "minimally invasive" OR "minimally invasive surgery" OR keyhole OR "minimal access") AND TITLE-ABS-KEY("resident*" OR "trainee*" OR "medical student*" OR "surgical education" OR "postgraduate training" OR "fellow*" OR novice OR "skill*" OR "train*" OR "educat*" OR psychomotor OR "technical skill*" OR "hand-eye" OR dexterity) AND PUBYEAR > 1999 AND PUBYEAR < 2027

n=2783

Web of Science:

TS=("Gamification" OR "Video Games" OR "Virtual Reality" OR gamificat* OR "game-based learning" OR "game based learning" OR "educational game*" OR "serious game*" OR "video game*" OR "computer game*" OR "simulation game*" OR "augmented reality" OR "mixed reality" OR "immersive" OR "head-mounted") AND TS=(laparoscop* OR laparoscopy OR "minimally invasive" OR "minimally invasive surgery" OR keyhole OR "minimal access") AND TS=("resident*" OR "trainee*" OR "medical student*" OR "surgical education" OR "postgraduate training" OR "fellow*" OR novice OR "skill*" OR "train*" OR "educat*" OR psychomotor OR "technical skill*" OR "hand-eye" OR dexterity) AND PY=2000-2026

n=1812

ClinicalTrials:

(surgeons OR resident OR residents OR trainee OR trainees OR "medical student" OR "medical students" OR "surgical trainee" OR "surgical trainees" OR dexterity) AND ("virtual reality" OR "augmented reality" OR "mixed reality" OR gamification OR "serious game" OR "serious games" OR "game-based" OR "video game" OR "video games" OR "computer simulation" OR simulation OR simulator OR "head mounted" OR "head-mounted display") AND (laparoscopy OR laparoscopic OR laparoscope OR "minimally invasive surgery" OR "minimally invasive") AND (education OR training OR "surgical education" OR "surgical skill" OR "surgical skills" OR "technical skill" OR "skill acquisition" OR psychomotor OR "hand-eye coordination")

n=69

**PRISMA-S Checklist**

| **Section/topic** | **#** | **Checklist item** | **Location(s) Reported** |
| --- | --- | --- | --- |
| **INFORMATION SOURCES AND METHODS** | | | |
| Database name | 1 | Name each individual database searched, stating the platform for each. | Page6，para4，sentence1 |
| Multi-database searching | 2 | If databases were searched simultaneously on a single platform, state the name of the platform, listing all of the databases searched. | Page6，para4，sentence4 |
| Study registries | 3 | List any study registries searched. | Page6，para4，sentence1 |
| Online resources and browsing | 4 | Describe any online or print source purposefully searched or browsed (e.g., tables of contents, print conference proceedings, web sites), and how this was done. | Page6，para4，sentence4 |
| Citation searching | 5 | Indicate whether cited references or citing references were examined, and describe any methods used for locating cited/citing references (e.g., browsing reference lists, using a citation index, setting up email alerts for references citing included studies). | Page6，para4，sentence5 |
| Contacts | 6 | Indicate whether additional studies or data were sought by contacting authors, experts, manufacturers, or others. | Page6，para4，sentence6 |
| Other methods | 7 | Describe any additional information sources or search methods used. | No |
| **SEARCH STRATEGIES** | | | |
| Full search strategies | 8 | Include the search strategies for each database and information source, copied and pasted exactly as run. | Page6，para4，sentence8 |
| Limits and restrictions | 9 | Specify that no limits were used, or describe any limits or restrictions applied to a search (e.g., date or time period, language, study design) and provide justification for their use. | In multiplemedia appendix 1 |
| Search filters | 10 | Indicate whether published search filters were used (as originally designed or modified), and if so, cite the filter(s) used. | Page6，para4，sentence7 |
| Prior work | 11 | Indicate when search strategies from other literature reviews were adapted or reused for a substantive part or all of the search, citing the previous review(s). | Page6，para4，sentence2 |
| Updates | 12 | Report the methods used to update the search(es) (e.g., rerunning searches, email alerts). | No |
| Dates of searches | 13 | For each search strategy, provide the date when the last search occurred. | Page6，para4，sentence2 |
| **PEER REVIEW** | | | |
| Peer review | 14 | Describe any search peer review process. |  |
| **MANAGING RECORDS** | | | |
| Total Records | 15 | Document the total number of records identified from each database and other information sources. | In multiplemedia appendix 1 |
| Deduplication | 16 | Describe the processes and any software used to deduplicate records from multiple database searches and other information sources. | Page6，para4，sentence9 |
|  |  |  |  |
| PRISMA-S: An Extension to the PRISMA Statement for Reporting Literature Searches in Systematic Reviews | | |  |
| Rethlefsen ML, Kirtley S, Waffenschmidt S, Ayala AP, Moher D, Page MJ, Koffel JB, PRISMA-S Group. | | |  |
| Last updated February 27, 2020. | |  |  |
